# *Lysimachia xiangxiensis* (Primulaceae), a new species from limestone area in Hunan Province, central China

**DOI:** 10.3897/phytokeys.140.47995

**Published:** 2020-02-24

**Authors:** Cun Mou, Yu Wu*, Liang Xiang, Xiao-Mei Xiang, Dai-Gui Zhang

**Affiliations:** 1 Hunan Forest Botanical Garden, Changsha 410116, Hunan, China Hunan Forest Botanical Garden Changsha China; 2 Key Laboratory of Cultivation and Protection for Non-Wood Forest Trees of Ministry of Education, Changsha 410004, Hunan, China Key Laboratory of Cultivation and Protection for Non-Wood Forest Trees of Ministry of Education Changsha China; 3 Key Laboratory of Non-Wood Forest Product of State Forestry Administration, Central South University of Forestry and Technology, Changsha 410004, Hunan, China Central South University of Forestry and Technology Changsha China; 4 College of Life Sciences, Hunan Normal University, Changsha 410081, Hunan, China Hunan Normal University Changsha China; 5 Jishou Dehang Scenic Area Management Office, Jishou 416000, Hunan, China Jishou Dehang Scenic Area Management Office Jishou China; 6 College of Biology and Environmental Sciences, Jishou University, Jishou 416000, Hunan, China Jishou University Jishou China

**Keywords:** *
Lysimachia
*, *L. xiangxiensis*, new species, taxonomy, western Hunan

## Abstract

A new species of *Lysimachia*, *L. xiangxiensis* (Primulaceae), is described and illustrated from western Hunan, central China. The species is similar to *L. melampyroides* in plant densely strigillose, leaves subglabrous adaxially, and flowers usually solitary in axils of upper leaves, but differs by the succulent leaves, the creeping or ascending stems 15–25 cm long, and the suborbicular to broadly elliptic corolla lobes. This new species is also supported by a molecular phylogenetic analysis of some *Lysimachia* species based on ITS sequence data.

## Introduction

The genus *Lysimachia* L., a large genera of Primulaceae s. l. (APG III 2009), consists of over 180 species of annual or perennial herbs (Hu and Kelso 1996). *Lysimachia* has a nearly cosmopolitan distribution, mainly occurring in the temperate and subtropical parts of the northern hemisphere, with a few species in Africa, Australia and South America (Hu and Kelso 1996, Liu et al. 2014a). Southwestern China and its neighboring region of Indochina Peninsula have an extremely high species diversity with ca. 130 species and have been considered to be the diversity center of the genus (Yan et al. 2017).

During our expedition in 2017 and 2019 to the Youshui River valley in western Hunan, China, an unusual population of *Lysimachia*, with the plants having revolute succulent leaves, caught our attention. After consulting the relevant literature (Chen et al. 1989, Hu and Kelso 1996, Yan and Hao 2012, Liu et al. 2014a, Liu et al. 2014b, Zhou et al. 2015, Wang et al. 2018) and checking relevant specimens, we determined that the population represents a new species. Additionally, the new species is supported by a molecular phylogenetic analysis of some *Lysimachia* species based on ITS sequence data.

## Materials and methods

### Taxon sampling and morphological analysis

The type specimens and fresh materials of the new species were collected from Huayuan County and Jishou City, Hunan Province, central China. Morphological observations and measurements were randomly made on flowering and fruiting plants. We examined related specimens kept in JIU and HUN and also specimen images in the online database of Chinese Virtual Herbarium (http://www.cvh.ac.cn) and JSTOR Global Plants (https://plants.jstor.org).

A total of 39 nuclear ribosomal ITS sequences for 34 species (Appendix S1) were downloaded from GenBank, following a study of *Lysimachia* (Zhang et al. 2011, Zhou et al. 2015). Two accessions of the putatively new species were sequenced for this study (GenBank Acc. No.: MN647744, MN647745). *Ardisia verbascifolia* Mez was selected as outgroup following Zhang et al. (2011). Voucher specimens of those specimens of the new species used for sequencing were deposited in JIU.

### Molecular analyses

Total genomic DNA of the two accessions of the putatively new species was isolated from silica gel-dried leaves using a modified cetyltrimethylammonium bromide procedure (Doyle and Doyle 1987). The ITS region was amplified and sequenced by method of Zhang et al. (2011).

Phylogenetic trees were constructed using maximum likelihood (ML) and Bayesian inference (BI). The models determined for the datasets using the Akaike information criterion (Burnham and Anderson 2003) as implemented in MrModeltest 2.3 (Nylander 2004). ML trees were generated in RAxML 7.2.6 (Stamatakis 2006) with 1000 bootstrap replicates. BI trees were inferred in MrBayes version 3.1.2 (Huelsenbeck and Ronquist 2001). Four chains, each starting with a random tree, were run for 1,000,000 generations with trees sampled every 1000 generations. The convergence of the two runs was accessed with the average standard deviation of split frequencies less than 0.01. After the first ca. 25% discarded as burn-in, the remaining trees were imported into PAUP* v.4.0b10 (Swofford 2002) and a 50% majority rule consensus tree was produced to obtain posterior probabilities (PP) of the clades.

## Results and discussion

### Morphological comparisons

According to the key in Hu and Kelso (1996), the new species is positioned to “Key 2” by flowers 5-merous, homomorphic, corolla yellow, anthers shorter than filaments, and further to “19a” by anthers distinctly dorsifixed (1b), inflorescences not paniculate(3b), stems more than 5 cm and leaves opposite (5b), corolla subfunnelform, filaments connate 1/3–1/2 into a tube (7b), flowers axillary and solitary or in terminal clusters with bracts leaflike (12b), inflorescences not capitate (17a), leaf blade not connate-perfoliate (18b), flowers solitary and axillary or in terminal racemes, plants strigillose (19a).

Morphologically, the new species is most similar to *L. melampyroides* R. Knuth in Engler with which it shares such features as the plants densely strigillose, leaves subglabrous adaxially, and flowers that are usually solitary in axils of upper leaves. However, the new species differs from *L. melampyroides* by the succulent leaves, the creeping or drooping stems 15–25 cm long, and the suborbicular to broadly elliptic corolla lobes. A morphological comparison between the new species and *L. melampyroides* is presented in Table 1.

**Table 1.** Morphological comparison between *Lysimachia xiangxiensis* sp. nov. and its similar species.

**Character**	***L. xiangxiensis* sp. nov.**	***L. melampyroides***
Stems	creeping or drooping.	erect or ascending.
Plant height	15–25 cm	15–50 cm
Petiole	not auriculate at base	dilated and auriculate at base
Blades of lower leaves	succulent, rhomboid-ovate to ovate, the basal 1 or 2 pairs scale-like	papery, ovate to linear-lanceolate
Blades of upper leaves	succulent, ovate to elliptic-lanceolate, 2–5.5 cm × 1–2.3 cm	papery, ovate to linear-lanceolate, 1.5–9 × 0.3–2.5 cm
Secondary veins	blurry or invisible on adaxial surface, slightly raising on abaxial surface	visible on both surfaces
Glandular dots on leaves	Absent	transparent, sparse
Corolla lobes	suborbicular to broadly elliptic, apex cuspidate or emarginated, 7–9 mm long and wide	obovate-elliptic, apex rounded, 6–7 × 4–6 mm
Calyx lobes	costa indistinct	costa distinct

### Phylogenetic position

**Figure 1.**
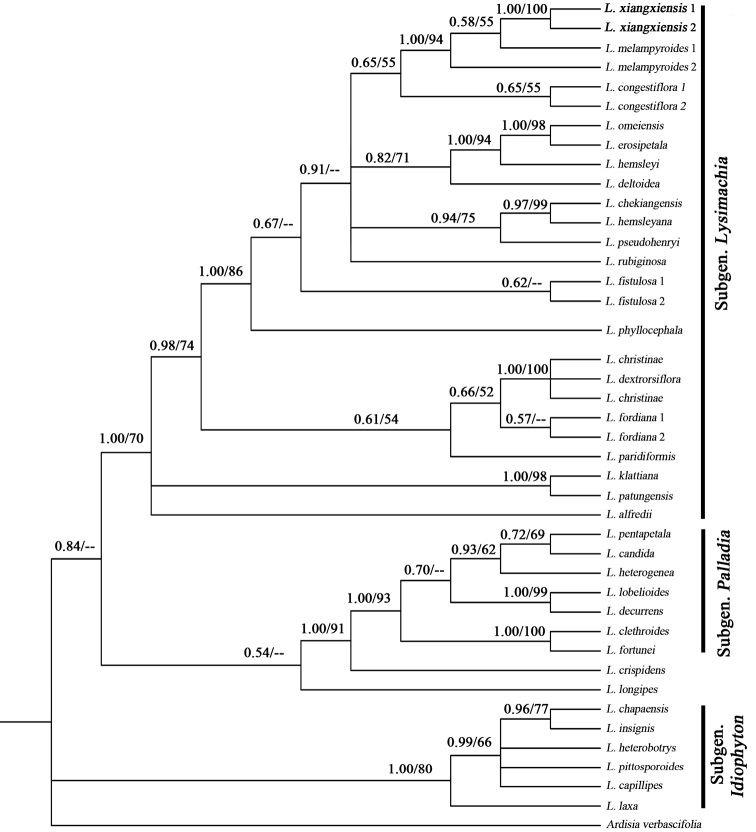
The phylogram of Bayesian inference (BI) tree from the ITS sequence data, showing the phylogenetic position of *Lysimachia xiangxiensis* sp. nov. (shown in bold). Values above the branches represent Bayesian posterior probabilities (PP) and bootstrap values (LP) for maximum likelihood, respectively; the dash (–) indicates LP < 50%.

The aligned lengths of ITS are 655 bp with gaps treated as missing data. BI and ML analyses produced similar topology and only the BI tree was presented in Figure 1. The phylogenetic results indicate that two samples of the new species were grouped together with a strong support (PP = 1.00, LP = 100%) and closely related to *L. melampyroides* (PP = 1.00, LP = 94%).

On the basis of the classification in Handel-Mazzetti (1928), Chen and Hu (1979) divided the genus into five subgenera as well as many series, Subgen. Lysimachia, Subgen. Palladia (Moench) Hand.-Mazz, Subgen. Idiophyton Hand. -Mazz., Subgen. Naumburgia (Moench) Klatt and Subgen. Heterostylandra (Hand.-Mazz.) Chen et C. M. Hu. In this topology, all *Lysimachia* species form three main clades: Subgen. Lysimachia (PP = 1.00, LP = 70%), Subgen. Palladia (PP = 1.00, LP = 93%) and Subgen. Idiophyton. (PP = 1.00, LP = 80%). In addition, *L. crispidens* (Hance) Hemsley in F. B. Forbes & Hemsley of Subgen. Heterostylandra is close to Subgen. Palladia (PP = 1.00, LP = 91%) and *L. longipes* Hemsley is assigned to Subgen. Lysimachia with weak supported (PP = 0.54) in a neutral position between Subgen. Lysimachia and *L. crispidens*. But classification of series are not well reflected in this analysis.

### Taxonomic treatment

#### Lysimachia xiangxiensis

Taxon classificationPlantaeEricalesPrimulaceae

D.G.Zhang & C.Mou, Y.Wu sp. nov.

EC01155A-F8FC-5F06-9EE6-87AEED57D257

urn:lsid:ipni.org:names:77206205-1

Figure 2 , 3 , 4

##### Type.

CHINA. Hunan Province, Huayuan County, Buchou Town, Da-long-dong, cliff of a valley, 28°19'06.42"N, 109°30'03.22"E, alt. 295 m, 26 August 2019, D. G. Zhang 0826075 (holotype: JIU!; isotype: JIU!).

##### Diagnosis.

The new species differs from *L. melampyroides* by the succulent leaves; the creeping or drooping stems (15–25 cm long); and the suborbicular to broadly elliptic corolla lobes.

##### Description.

Terrestrial, perennial herbs. Rhizome brown, reduced to a small tuber or rarely creeping, with sparse fibrous roots. ***Stems*** creeping or drooping on cliffs, 15–25 cm long, clustered, branched at base, unbranched or rarely branched from the middle, terete, purple-red, densely strigillose, the internodes usually 3–7 cm long. ***Leaves*** petiolate, opposite. Petioles 5–7 mm long, with a furrow on adaxial side, green or purple-red, strigillose. Leaf blade succulent; blade of lower leaves rhomboid-ovate to ovate, with 1 or 2 pairs of basal leaves scalelike (much smaller); blade of upper leaves ovate to elliptic-lanceolate, 2–5.5 cm × 1–2.3 cm, base cuneate, apex acuminate or acute to subobtuse, margin entire and revolute, adaxially dark green, shiny, subglabrous, abaxially purple-red (in arid places) or light green (in moist places), densely strigillose along the midrib, not glandular on both surfaces; secondary veins 3–4 pairs, blurry or invisible adaxially, slightly raising abaxially, veinlets invisible. ***Flowers*** bisexually, solitary in axils of upper leaves, occasionally in terminal racemes with bractlike leaves. ***Pedicels*** 1.5–3 cm long, gradually reduced toward stem apex, purple-red or light purple-red, densely strigillose, recurved in fruit. ***Calyx*** lobes 5, rarely 6, persistent, lanceolate with indistinct costa, 6–8 mm × 1.5–2 mm, apex acuminate-subulate, inside glabrous and with 3–4 veins, outside purple-red or green, densely strigillose. ***Corolla*** yellow, tube 1–2 mm long, actinomorphic, contorted; lobes 5, 7–9 mm × 7–9 mm, suborbicular to broadly elliptic, apex cuspidate or rounded, erose above the middle. ***Stamens*** 5, yellow, opposite to corolla lobes; ***filaments*** connate basally into a tube ca. 2.5 mm high, free parts 3.5–4.5 mm; ***anthers*** ca. 2 mm long, dorsifixed, opening by lateral slits. ***Style*** ca. 6 mm long, apex slightly expanded, strigillose on lower part. ***Ovary*** cylindrical, ca. 1.5 cm in diam., strigillose on apex, superior. Capsule brown, subglobose, 3–4 mm in diam., densely strigillose, dehiscing by valves. Seeds small, black, angular, papillate.

##### Phenology.

Flowering May–June, fruiting July–August.

##### Distribution and habitat.

**Figure 2.**
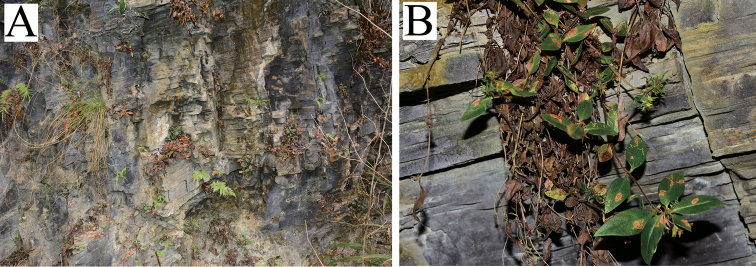
*Lysimachia xiangxiensis* sp. nov. in the wild **A** habitat (dry limestone cliff) **B** stems drooping on the cliff.

This new species is currently known from Huayuan County and Jishou City in western Hunan Province, central China. It usually grows on limestone cliffs in valleys (Figure 2), and is associated with e.g. *Eriophorum comosum* (Wallich) Nees in Wight, *Pteris vittata* Linnaeus, *Pteris deltodon* Baker, and *Dryopteris* sp.

**Figure 3.**
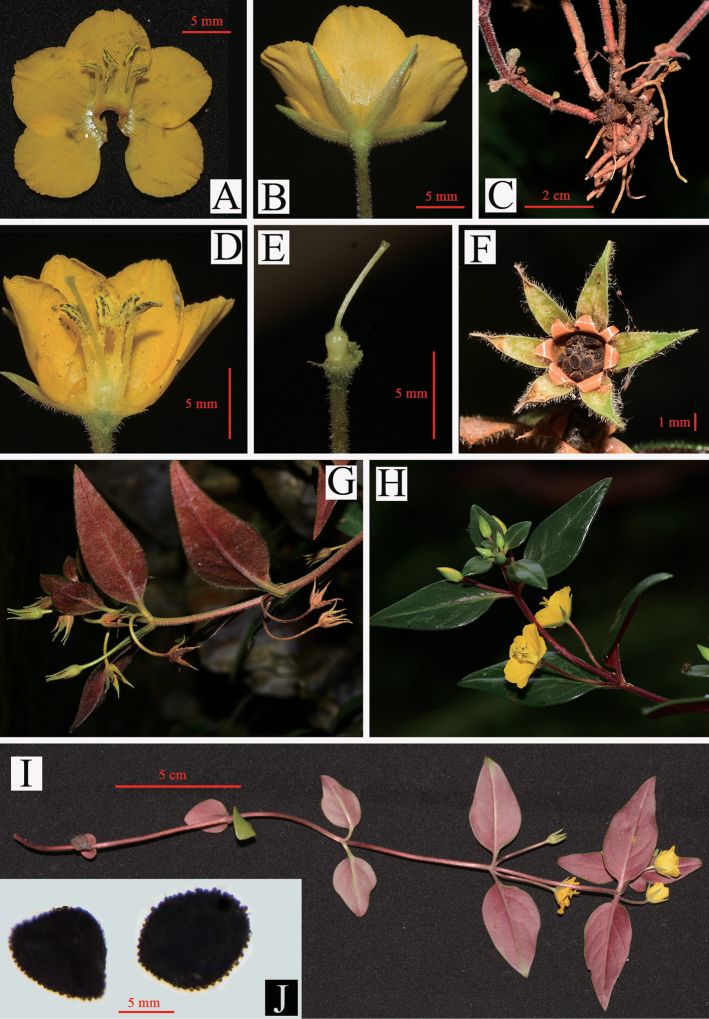
*Lysimachia xiangxiensis* sp. nov. in the field **A** corolla opened, showing the suborbicular lobes **B** flower (lateral view), showing the lanceolate calyx lobes indistinctly costate **C** proximal stems and underground part, showing stems clustered, rhizome, sparse fibrous roots, and 1 or 2 pairs of scalelike basal leaves **D** longitudinal section of flower, showing filaments connate basally into a tube **E** pistil, showing strigillose hairs on apex of ovary and base of style **F** dehiscent capsule **G** plant in fruiting, showing the recurved pedicels **H** plant in ﬂowering, showing the solitary flowers in axils of upper leaves **I** plant in ﬂowering, showing the reduced basal leaves **J** papillate seeds

**Figure 4.**
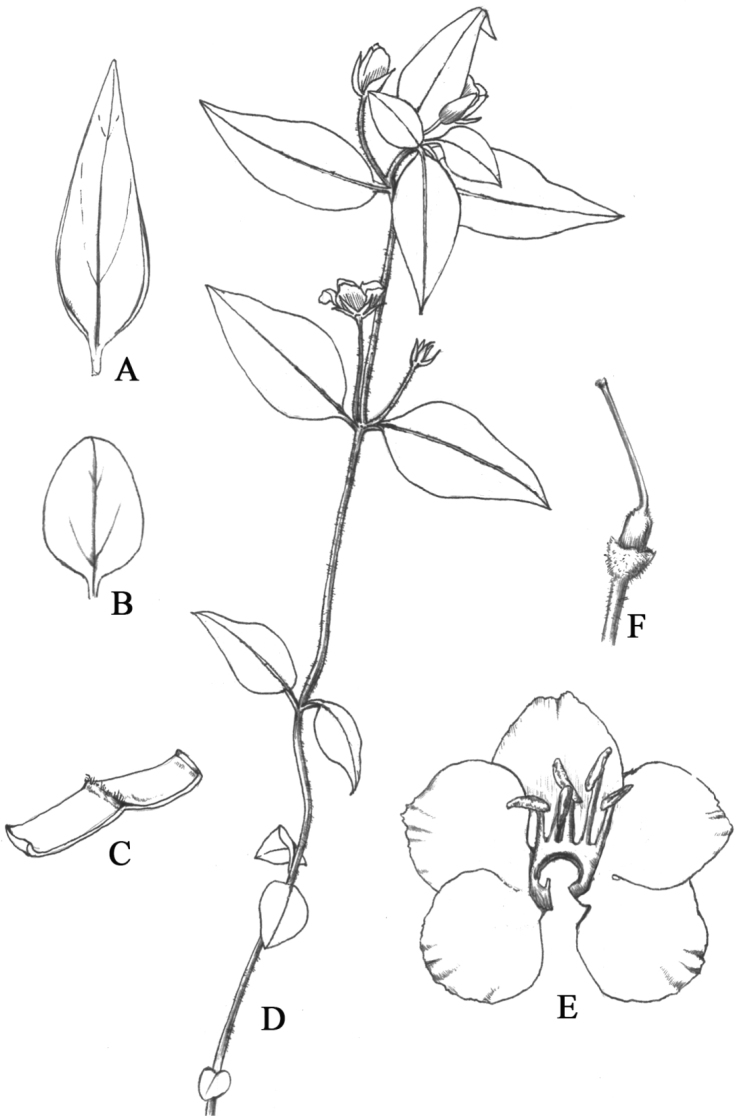
*Lysimachia xiangxiensis* sp. nov. **A** upper stem leaf (abaxial surface), showing revolute margins **B** lower stem leaf (adaxial surface) **C** portion of a leaf (abaxial surface), showing the revolute margins and strigillose midrib **D** plant in ﬂowering **E** corolla lobes **F** pistil.

##### Etymology.

The specific epithet “*xiangxiensis*”, literally meaning western Hunan, refers to the Xiangxi Tujia and Miao Autonomous Prefecture in central China, to which Huayuan County and Jishou City belong. The Chinese name of the *Lysimachia xiangxiensis* is xiang xi guo lu huang in Pinyin.

##### Conservation status.

*Lysimachia xiangxiensis* usually grows on limestone cliffs in valleys so we suggest its placement in the Data Deficient category of IUCN (2017)

##### Additional collection.

CHINA. Hunan Province, Jishou City, Aizhai Town, National Forest Park, cliff of a valley, 31 May 2019, Y. Wu 0531001(paratype, JIU!).

## Supplementary Material

XML Treatment for Lysimachia xiangxiensis

## Appendix

**Appendix S1.** Accessions of the genus *Lysimachia* L. examined in this study.

**Taxon**	**GenBank Acc. No.**	**Voucher**	**Locality**
*L. alfredii* Hance	JN638405	Hao394	Lianping, Guangdong, China
*L. candida* Lindley	JF976885	Ge2010001	Yangchun, Guangdong, China
*L. capillipes* Hemsley in F. B. Forbes & Hemsley	JF976886	Y2009200	Jiujiang, Jiangxi, China
*L. chapaensis* Merrill	JF976888	GBOWS878	Hekou, Yunnan, China
*L. chekiangensis* C. C. Wu	JF976891	Y2009263-1	Longquan, Zhejiang, China
*L. christiniae* Hance	JF976894	Y2009244	Lin'an, Zhejiang, China
	JF976896	Y2009209	Jiujiang, Jiangxi, China
*L. clethroides* Duby in A. de Candolle	JF976899	Y2009157	Tongbai, Henan, China
*L. congestiflora* Hemsley in F. B. Forbes & Hemsley	JF976902	GBOWS262	Malipo, Yunnan, China
	JF976903	Y2009266	Longquan, Zhejiang, China
*L. crispidens* (Hance) Hemsley in F. B. Forbes & Hemsley	JF976906	Hao212	Yichang, Hubei, China
*L. decurrens* G. Forster	JF976908	GBOWS1234	Hekou, Yunnan, China
*L. deltoidea* Wight	JF976909	GLM081121	Zhongdian, Yunnan, China
*L. dextrosiflora* X. P. Zhang, X. H. Guo & J. W. Shao	JF976913	Y2009265-1	Longquan, Zhejiang, China
*L. erosipetala* F. H. Chen & C. M. Hu	JF976914	Y2010037-2	Emeishan, Sichuan, China
*L. fistulosa* Handel-Mazzetti	JF976916	Ning20101	Jinggangshan, Jiangxi, China
	JF976917	Ye et al. 3561	Lianshan, Guangdong, China
	JF976919	Y2009285	Ruyuan, Guangdong, China
	JF976920	Ye et al. 3940	Lianshan, Guangdong, China
*L. fortune* Maximowicz	JF976925	Y2009195	Jinggangshan, Jiangxi, China
*L. hemsleyana* Maximowicz ex Oliver	JF976932	Guo20001	Ningguo, Anhui, China
*L. hemsleyi* Franchet	JF976935	Hao713	Huili, Sichuan, China
*L. heterobotrys* F. H. Chen & C. M. Hu	JF976936	Y2010053-2	Ningming, Guangxi, China
*L. heterogenea* Klatt	JF976939	Y2009199	Jiujiang, Jiangxi, China
*L. insignis* Hemsley	JF976945	Hao245	Napo, Guangxi, China
*L. klattiana* Hance	JF976947	Y2010014-1	Tongbai, Henan, China
*L. laxa* Baudo	JF976949	Han longran6	Puer, Yunnan, China
*L. lobelioides* Wallich in Roxburgh	JF976951	Hao303	Menglian, Yunnan, China
*L. longipes* Hemsley	JF976952	Guo xinhu200012	Shitai, Anhui, China
*L. melampyroides* R. Knuth in Engler	JF976955	Dengyunfei15945	Xinning, Hunan, China
	JF976956	Lichanghan8174	Shangzhi, Hunan, China
*L. omeiensis* Hemsley	JF976958	Y2010033	Emeishan, Sichuan, China
*L. paridiformis* Franchet	JF976962	Y2010044	Emeishan, Sichuan, China
*L. patungensis* Handel-Mazzetti	JF976964	Ye et al. 3851	Lianshan, Guangdong, China
*L. pentapetala* Bunge	JN638407	Y2010013-1	Tongbai, Henan, China
*L. phyllocephala* Handel-Mazzetti	JF976969	Y2010030	Emeishan, Sichuan, China
*L. pittosporoides* C. Y. Wu	JF976970	Hao248	Malipo, Yunnan, China
*L. rubiginosa* Hemsley in F. B. Forbes & Hemsley	JF976972	Hao419	Dujiangyan, Sichuan, China
*Lysimachia xiangxiensis* D.G.Zhang & C.Mou, Y.Wu, sp. nov.	MN647745	Y. Wu 0531001	Jishou, Hunan, China
	MN647744	D. G. Zhang 0826075	Huayuan, Hunan, China
*Ardisia verbascifolia* Mez	JN638408	GBOWS1216	Hekou, Yunnan, China
